# Integrated metabolomic and transcriptomic analyses of the parasitic plant *Cuscuta japonica* Choisy on host and non-host plants

**DOI:** 10.1186/s12870-022-03773-9

**Published:** 2022-08-08

**Authors:** Chenglin Guo, Liuyan Qin, Yongling Ma, Jianlin Qin

**Affiliations:** 1 Plant Protection Research Institute, Guangxi Academy of Agricultural Science/ Key Laboratory of Green Prevention and Control on Fruits and Vegetables in South China Ministry of Agriculture and Rural Affairs/Guangxi Key Laboratory of Biology for Crop Diseases and Insect Pests, Nanning, 530007 China; 2 Biotechnology Research Institute, Guangxi Academy of Agricultural Sciences, Nanning, 530007 China

**Keywords:** *Cuscuta japonica* Choisy, Host plant, Non-host plant, Parasitism, Metabolome, Transcriptome, Phenylpropanoid biosynthesis

## Abstract

**Background:**

*Cuscuta japonica* Choisy (Japanese dodder) is a parasitic weed that damages many plants and affects agricultural production. The haustorium of *C. japonica* plays a key role during parasitism in host plants; in contrast, some non-host plants effectively inhibit its formation. However, the metabolic differences between normal dodder in host plants and dodder inhibition in non-host plants are largely unknown. Here, we utilized an integrative analysis of transcriptomes and metabolomes to compare the differential regulatory mechanisms between *C. japonica* interacting with the host plant *Ficus microcarpa* and the non-host plant *Mangifera indica*.

**Results:**

After parasitization for 24 h and 72 h, the differentially abundant metabolites between these two treatments were enriched in pathways associated with α-linolenic acid metabolism, linoleic acid metabolism, phenylpropanoid biosynthesis, and pyrimidine metabolism. At the transcriptome level, the flavor biosynthesis pathway was significantly enriched at 24 h, whereas the plant–pathogen interaction, arginine and proline metabolism, and MARK signaling-plant pathways were significantly enriched at 72 h, based on the differentially expressed genes between these two treatments. Subsequent temporal analyses identified multiple genes and metabolites that showed different trends in dodder interactions between the host and non-host plants. In particular, the phenylpropanoid biosynthesis pathway showed significant differential regulation between *C. japonica* in host and non-host plants.

**Conclusions:**

These results provide insights into the metabolic mechanisms of dodder–host interactions, which will facilitate future plant protection from *C. japonica* parasitism.

**Supplementary Information:**

The online version contains supplementary material available at 10.1186/s12870-022-03773-9.

## Background

*Cuscuta japonica* Choisy, commonly known as the Japanese dodder, is an annual or perennial holoparasitic plant that mainly endangers woody plants, such as tropical and subtropical fruit trees, economic trees, and ornamental trees [59]. The plant has become a major hazard to gardens and forest greening in Guangxi, southern China, because of its wide host range and strong vitality and has proven difficult to control. *Cuscuta japonica* is an all-parasitic seed plant that absorbs water, nutrients, and other macromolecules from the host plant through its haustorium, which is a special absorption organ [41]. The haustorium develops from the haustorial primordium, which differentiates from the cortical cells of the stem in dodders [53, 60]. When seedlings contact the host surface, the upper haustorium (outside the host tissue) develops in the middle cortex of the stem. Once the dodder haustorium forms a parasitic relationship with the host, it is difficult to control it chemically [12]. Some chemical herbicides have poor selectivity between host and parasitic plants [42]; despite controlling the harm caused by the parasite, they often cause serious damage to host plants. Some selected chemicals can control independent growth of dodder stems but are not effective on the haustorium and its adjacent tissues. The haustorium rapidly absorbs nutrients from the host plant, regerminates, and continues to parasitize [49]. Thus, screening selective agents with strong effects on the haustorium is key to the efficient control of dodder and can provide ideas for the rational design of high-efficiency control agents through the investigation of the mechanism of haustorial inhibition and by clarifying the action target. This may provide an innovative breakthrough in ecological and efficient dodder control.

*Cuscuta* has a broad host spectrum and several possible plant “victims.” However, few plants have defense mechanisms that can successfully prevent *Cuscuta* infestation [27]. At present, the few available studies on the mechanism of inhibition of haustoria formation in *C. japonica* have mainly focused on the stress response and resistance of resistant hosts. Werner et al. [52] found that when seedlings were exposed to a host of resistant tomato plants, tomato stem cortical cells exhibited a hypersensitivity reaction. The epidermal, subcutaneous, and collenchyma tomato cells are elongated, and the cortical parenchyma cells differentiate under the contact point to form hemispherical and ladder boundary tissues, forming the original structural defense line [4]. Hegenauer et al. [20] found that a glycine-rich protein of the parasite cell wall, Crip21, specifically binds and activates a membrane-bound immune receptor in tomato, cuscuta receptor 1 (CuRe1), leading to defense responses in resistant hosts. Moreover, resistant hosts, including sweet potato and tomato, can produce degrading enzymes in cell walls and inhibit the penetration of haustorium into plant tissues [3]. Masanga et al. [40] found that *Mangifera indica* successfully mounted a post-attachment resistance response to dodder. In summary, in direct contact with haustoria, epidermal tomato cells elongate and die following a hypersensitive response, whereas a secondarily modified tissue is formed in the hypodermis to protect against haustoria penetration [23].

Because of the vigorous metabolism of haustorium tissue cells, the carbon and nitrogen compounds and organic acids entering the haustorium from the host plant can be processed into more easily absorbed substances for the use of parasitic plants [47], and the presence of abundant RNA near haustoria also promotes RNA transfer between parasitic plants and hosts [46]. *TvPirin*, which responds to haustoria-inducing factors, was identified in *Triphysaria versicolor* through transcriptome research. Interference with *TvPirin* using RNAi significantly reduced haustorium formation [6]. Moreover, protein and RNA transfer between parasitic plants and hosts occur through the haustorium. Jiang et al. [26] found that phosphinothricin acetyltransferase could be transferred from the host to the dodder plant, resulting in herbicide resistance. In another experiment, a specific 277-bp fragment reverse repeat of mannose 6-phosphate reductase mRNA was overexpressed in tomatoes, after which the siRNA was detected in its R1 generation. In branch columns grown on transgenic tomatoes, the expression of mannose 6-phosphate reductase mRNA decreased by 60–80%, and the level of mannitol decreased significantly, also proving that siRNA can be transferred through this parasitic relationship [5].

We hypothesized that non-host plants might be resistant to dodder by interfering with the normal metabolism of dodder during haustoria formation. However, most studies have explored the causes of haustorial inhibition based on macro-tissue structure or physiological changes, without clear targets and mechanisms. With the development of omics technologies, the metabolome and transcriptome have become important research tools for investigating plant–plant interactions [24, 32]. In the present study, an integrative analysis of transcriptomes and metabolomes was performed between *C. japonica* parasitizations on host and non-host plants to investigate the regulatory mechanism of the interaction between *C. japonica* and host plants. After parasitization, the differentially abundant metabolites (DAMs) and differentially expressed genes (DEGs) of *C. japonica* were enriched in pathways associated with fatty acid metabolism, phenylpropanoid biosynthesis, and pyrimidine metabolism. In particular, the phenylpropanoid biosynthesis pathway showed significant differential regulation between *C. japonica* in host and non-host plants. These results provide insights into the metabolic mechanisms of dodder–host interactions.

## Results

### Comparison of the phenotype of *C. japonica* on host and non-host plants

**Fig. 1:**
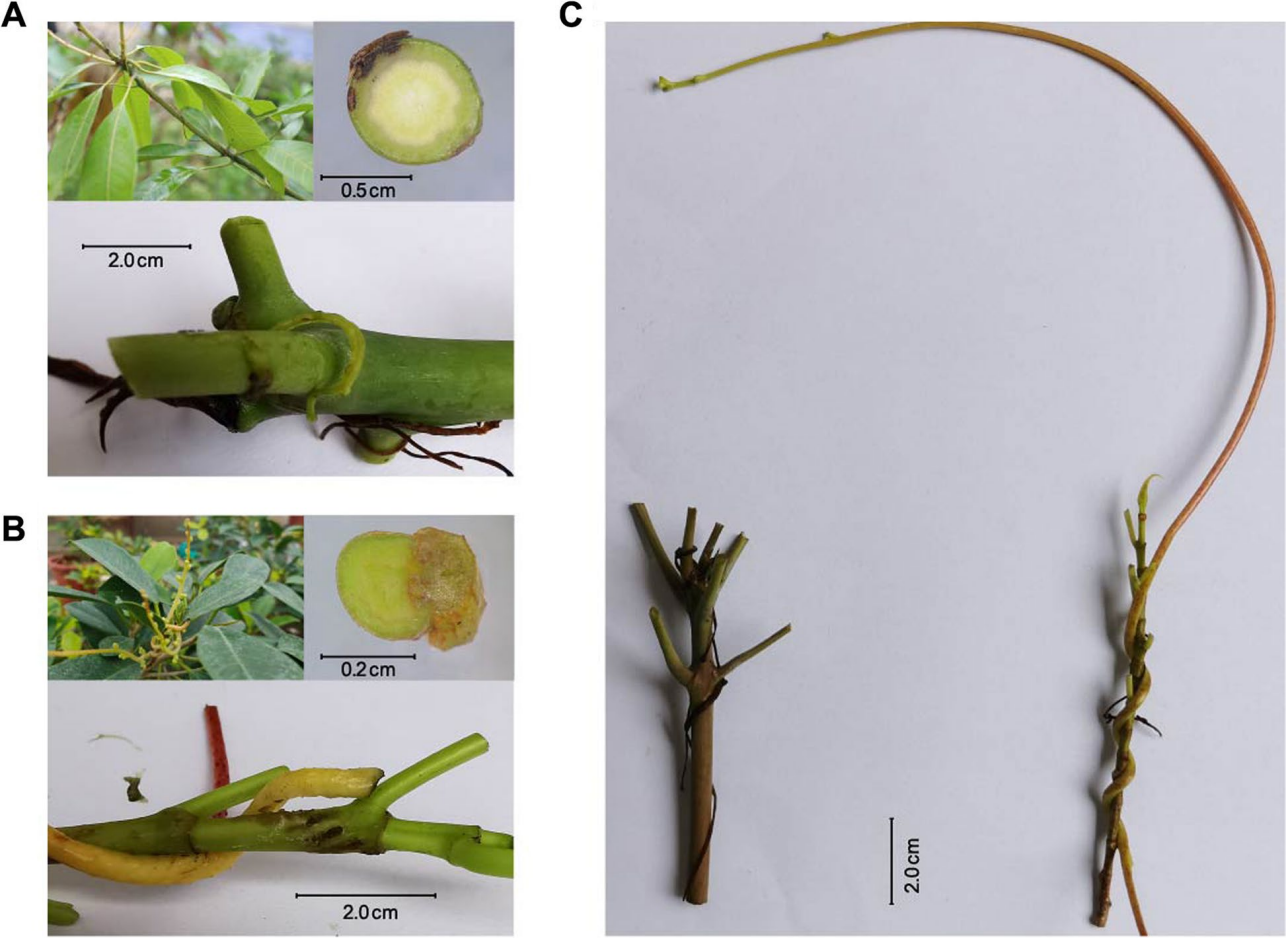
*Cuscuta japonica* planted on non-host *Mangifera indica* (**A**) and host *Ficus microcarpa* ‘Golden Leaves’ (**B**) including close-up of a parasitic vine entwining and cross section through an infection site. **C***C. japonica* attached to non-host (left) and host (right) plants after 15 days

Although *C. japonica* lacks leaves and roots, it can grow several stems to twist around the host and develop haustoria for host attachment and intrusion, which can extract inorganic compounds, nutrients, and water from the host. *Cuscuta japonica* exhibited distinct morphological features after attachment to *F. microcarpa* ‘Golden Leaves’ (host) and *M. indica* (non-host) plants. We observed that although the seedlings of dodder warped around *M. indica*, the haustorial roots did not fully penetrate the bark of *M. indica* (Fig. 1A). In contrast, haustorial growth was stimulated by the interaction between the dodder stem and host, as indicated by the retention of the green coloration of the dodder stem (Fig. 1B). The stem color of the dodder planted in the non-host became black, and the dodder eventually died after 15 d (Fig. 1C). These results suggest that *M. indica* can prevent the process that dodder absorbs nutrients through haustoria compared with *F. microcarpa* cv. Golden Leaves.

### Analysis of the metabolome during parasitization

**Fig. 2:**
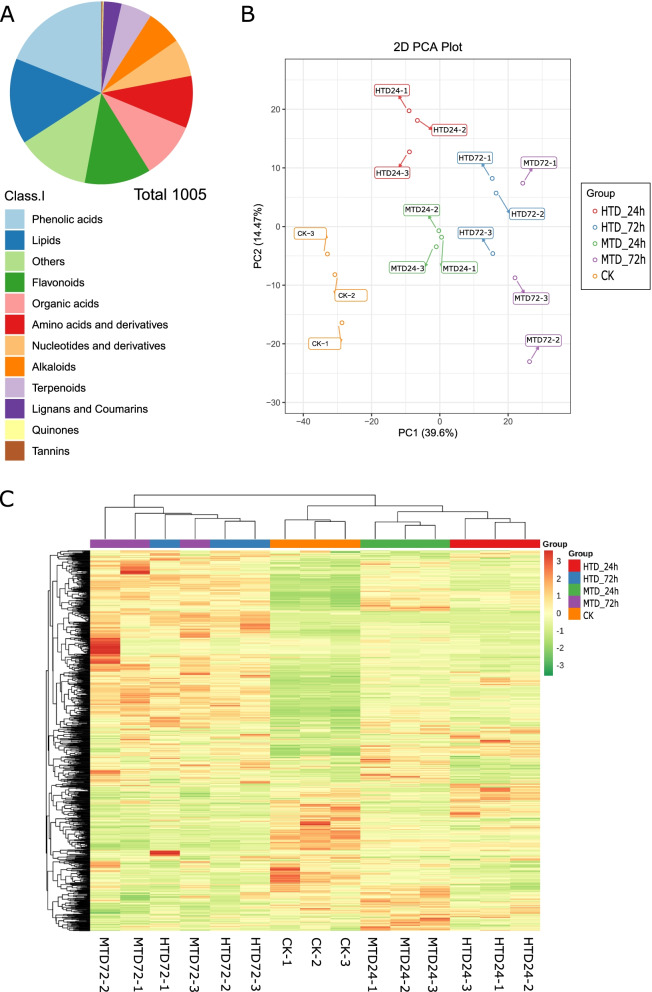
Distribution of identified metabolite classes (**A**); principal component analysis of samples (**B**); and heatmap of differentially accumulated metabolites in each sample (**C**). MTD, dodders attached to the stems of non-host plant; HTD, dodders attached to the stems of host plant

Significant differential phenotypes of haustoria formed in host and non-host plants suggest differential concentrations of metabolites in the stock. Therefore, we performed metabolome analysis using ultraperformance liquid chromatography–mass spectrometry (UPLC–MS) to obtain the metabolome profile of *C. japonica* and detected 1005 metabolites, which were classified into 12 classes, including 190 phenolic acids, 153 lipids, 130 others, 118 flavonoids, 100 organic acids, 93 amino acids and derivatives, 67 nucleotides and derivatives, 63 alkaloids, 55 terpenoids, 32 lignans and coumarins, 2 quinones, and 2 tannins (Table S1). These results suggest that phenolic acids, lipids, and flavonoids are the major metabolites of *C. japonica* during haustoria development (Fig. 2A).

To evaluate the metabolome differences between groups and variation status among the three replicates, we performed principal component analysis (PCA) of all samples. The PCA plot showed that initial seedling samples clearly distinguished between samples after 24 h and 72 h in the host (HTD_24h and HTD_72h) and non-host (MTD_24h and MTD_72h) plants, indicating large differences in metabolite concentrations in *C. japonica* (Fig. 2B). The replicates were clustered together in the PCA, suggesting low variability in the metabolome profile. The heatmap illustrates the 15 samples divided into two major clusters based on the ion abundance. One cluster contained initial seedling samples and MTD_24h and HTD_24h, and another cluster included MTD_72h and HTD_72h (Fig. 2C). Accordingly, more metabolites in *C. japonica* were relatively abundant 72 h after attachment (haa) than at 0 and 24 haa.

**Fig. 3:**
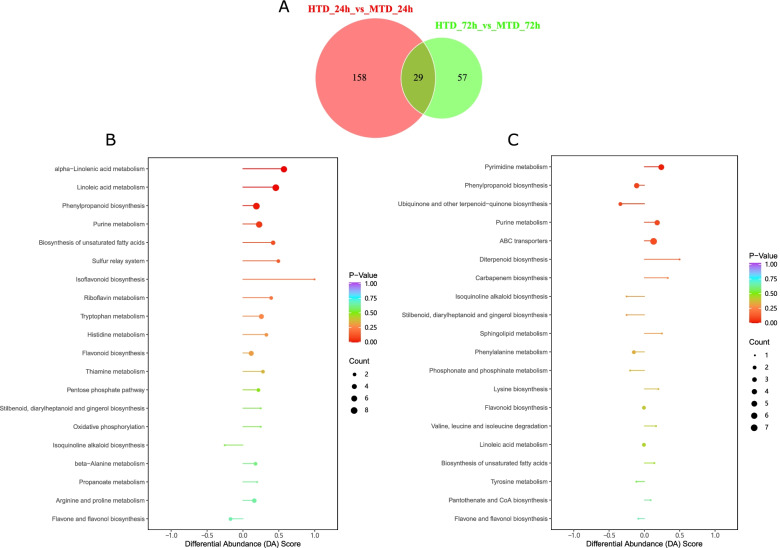
Number of differentially accumulated metabolites (**A**); pathway enrichment analysis for differentially abundant metabolites at 24 h (**B**); at 72 h (**C**). MTD, dodders attached to the stems of non-host plant; HTD, dodders attached to the stems of host plant

To explore metabolite differences in *C. japonica* attached to the two host types, we compared the abundance of metabolites at 24 h and 72 h. The abundance of phenolic acids in the host plants was lower than that in the non-host plants at 24 h. At 72 h, we found that nucleotides and derivatives, amino acids and derivatives, and phenolic acids were the top three differential classes based on their abundances (Tables S2 and S3; Fig. S1A, B). Moreover, we obtained 187 DAMs at 24 h and 86 DAMs at 72 h in the comparison between HTD and MTD, with threshold values of variable importance in projection (VIP) score ≥ 1 and fold change ≥2 (Fig. 3A). A total of 29 DAMs were common between 24 h and 72 h, suggesting a large variation in DAMs present in the haustoria of *C. japonica* at 24 h compared with metabolite profile alteration at 72 h. Furthermore, we used a differential abundance score to detect global changes in metabolites based on the Kyoto Encyclopedia of Genes and Genomes (KEGG) pathway enrichment analysis. α-linolenic acid metabolism, linoleic acid metabolism, and phenylpropanoid biosynthesis pathways were significantly enriched, containing multiple upregulated DAMs in MTD at 24 h compared with HTD at 24 h (Fig. 3B). Similarly, pyrimidine metabolism was significantly enriched by multiple upregulated DAMs in the MTD at 72 h compared with the HTD at 72 h (Fig. 3C).

### Analysis of transcriptome during parasitism

**Fig. 4:**
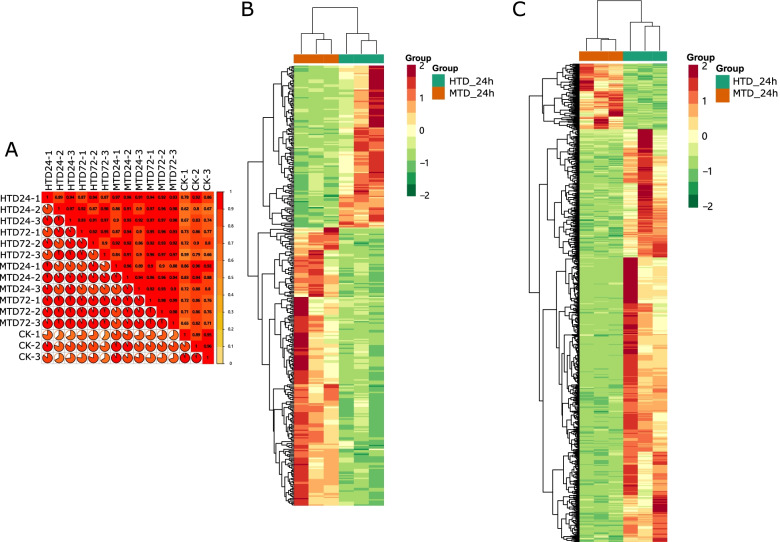
Gene expression analysis. Correlation across samples according to gene expression profiles (**A**). Differentially expressed genes in host and non-host plants at 24 h after attachment (**B**), Differentially expressed genes in hosts compared with those in non-hosts at 72 h after attachment (**C**). MTD, dodders attached to the stems of non-host plant; HTD, dodders attached to the stems of host plant

RNA was extracted from the samples used for metabolomic analysis and analyzed using RNA-seq. The number of raw reads among the samples ranged from 44 900 376 to 67 803 036. After removing low-quality reads, we obtained a total of 122.39 Gb of clean data, with an average of 6 Gb for each sample. The Q30 score of all products was greater than 92%, indicating the high quality of gene sequencing results for downstream analysis (Table S4). A total of 212 110 assembled unigenes with an average length of 726 bp were aligned to multiple databases, including KEGG, NCBI non-redundant (nr), Swiss-Prot, Gene Ontology, Clusters of Orthologous Groups/EuKaryotic Orthologous Groups (COG/KOG), Pfam, and Trembl to annotate the function of unigenes, of which 95 314 unigenes were mapped in at least one database. The Pearson’s correlation between replicates ranged from 0.89 to 0.97, suggesting that the transcriptome results were reliable and stable (Fig. 4A). A total of 348 DEGs at 24 h and 1123 DEGs at 72 h of pairwise samples were identified by threshold values of absolute log2 fold change (FC) value ≥1 and adjusted *P*-value < 0.05, respectively (Tables S5 and S6). Among these DEGs, 220 and 154 genes were upregulated, whereas 128 and 969 genes were downregulated in pairwise comparisons. Interestingly, when comparing HTD to MTD at 24 h, most of the upregulated genes belonged to the MTD samples. In contrast, at 72 h, most of the upregulated genes belonged to the HTD samples, suggesting biological function variation between HTD and MTD during haustoria development (Fig. 4B, C).

**Fig. 5:**
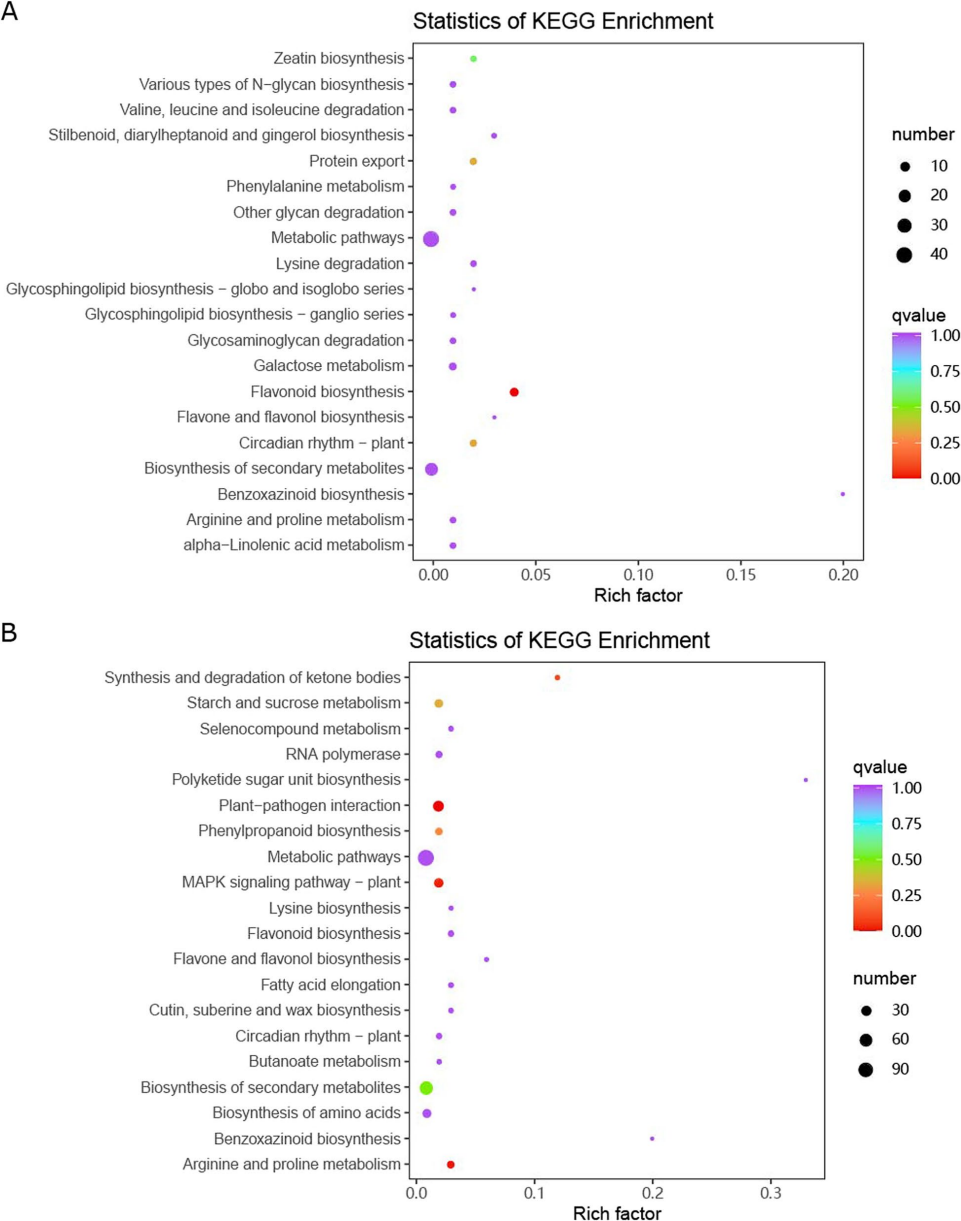
KEGG pathway enrichment analysis. DEGs generated by the comparison of HTD and MTD at 24 h (**A**). DEGs produced by HTD vs MTD at 72 h (**B**). We obtained permission to use the KEGG software from the Kanehisa laboratory. MTD, dodders attached to the stems of non-host plant; HTD, dodders attached to the stems of host plant; DEGs, differentially expressed genes

To systematically explore the biological functions of DEGs potentially involved in *C. japonica* after attachment, we used 348 and 1123 DEGs generated by pairwise comparisons at 24 h and 72 h, respectively, for KEGG pathway enrichment analysis. Flavonoid biosynthesis was significantly enriched by DEGs at 24 h, whereas three pathways, including plant–pathogen interaction (ko04626), arginine and proline metabolism (ko00330), and MARK signaling pathway plant (ko04016), were identified by KEGG analysis of HTD versus MTD at 72 h (Fig. 5).

### Differential trends of metabolites and genes in *C. japonica* on host and non-host plants

A total of 467 and 491 metabolites in *C. japonica* were found to be significantly upregulated and downregulated, respectively, during haustoria formation. To explore the pattern of metabolite accumulation, all metabolites in the HTD and MTD were classified into eight types of clusters using the STEM algorithm. The three clusters in each group, including profiles 0, 6, and 7, were significantly enriched by metabolites based on a *P* ≤ 0.05 (Fig. S2A, B). The trend in metabolite accumulation in the HTD and MTD during parasitism showed similar changes (Fig. S2C, D). For instance, metabolite levels in profile 6 of both groups showed a rapid increase from 0 to 24 haa, but slightly changed between 24 haa and 72 haa. Metabolite levels in profile 7 displayed a continuous increase, whereas those in profile 0 exhibited a downward trend from 0 to 72 haa. These results indicated that the metabolites involved in the significant profiles play an important role after attachment.

**Fig. 6:**
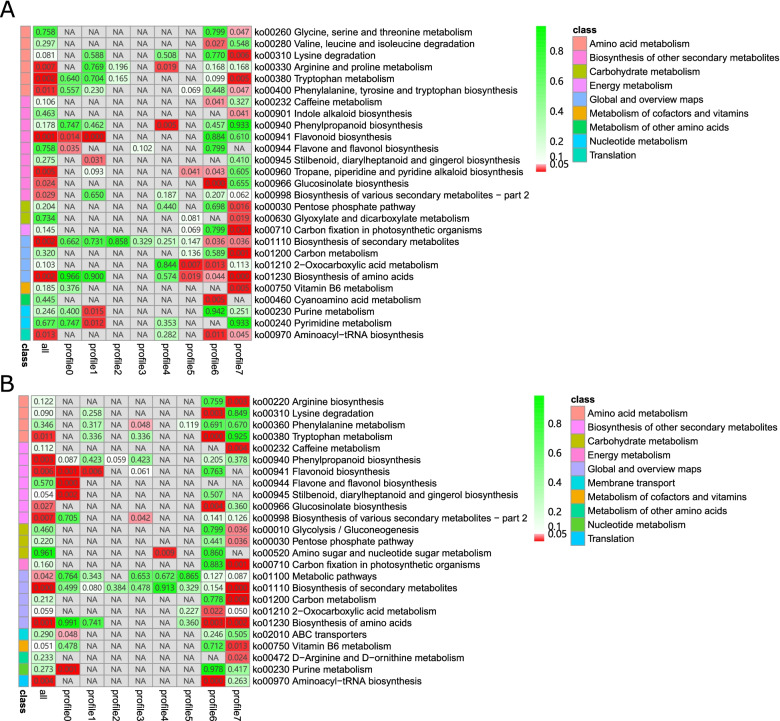
KEGG pathway enrichment analysis for metabolites in trend profiles of the HTD (**A**) and MTD (**B**) groups. We obtained permission to use the KEGG software from the Kanehisa laboratory. MTD, dodders attached to the stems of non-host plant; HTD, dodders attached to the stems of host plant

To evaluate the biological processes associated with the metabolites, we performed a KEGG enrichment analysis for the different metabolites involved in the profiles. Overall, 27 and 25 KEGG pathways were associated with all profiles in HTD and MTD, respectively, most of which belonged to significant profiles (Fig. 6). ABC transporters (ko02010), glycolysis/gluconeogenesis (ko00010), amino sugar and nucleotide sugar metabolism (ko00520), D-arginine and D-ornithine metabolism (ko00472), phenylalanine metabolism (ko00360), and arginine biosynthesis (ko00220) were specifically enriched pathways for metabolites in the trend profiles of MTD. In contrast, glycine, serine, and threonine metabolism (ko00260); valine, leucine, and isoleucine degradation (ko00280); arginine and proline metabolism (ko00330); phenylalanine, tyrosine, and tryptophan biosynthesis (ko00400); indole alkaloid biosynthesis (ko00901); troponin, piperidine, and pyridine alkaloid biosynthesis (ko00960); glyoxylate and dicarboxylate metabolism (ko00630); cyanoamino acid metabolism (ko00460); and pyrimidine metabolism (ko00240) were unique pathways for HTD. We also found that amino acid biosynthesis (ko01230) belonged to profiles 6 and 7 in both groups. Moreover, purine metabolism (ko00230) and metabolites involved in stilbenoid, diarylheptanoid, and gingerol biosynthesis (ko00945), belonging to profile 0 of MTD, showed a continuous decline, whereas ko00230 generated by profile 1 of HTD showed accumulation from 0 to 24 haa and remained stable between 24 haa and 72 haa, suggesting that metabolites of the common KEGG pathway between HTD and MTD contained different accumulation patterns based on haustoria development.

**Fig. 7:**
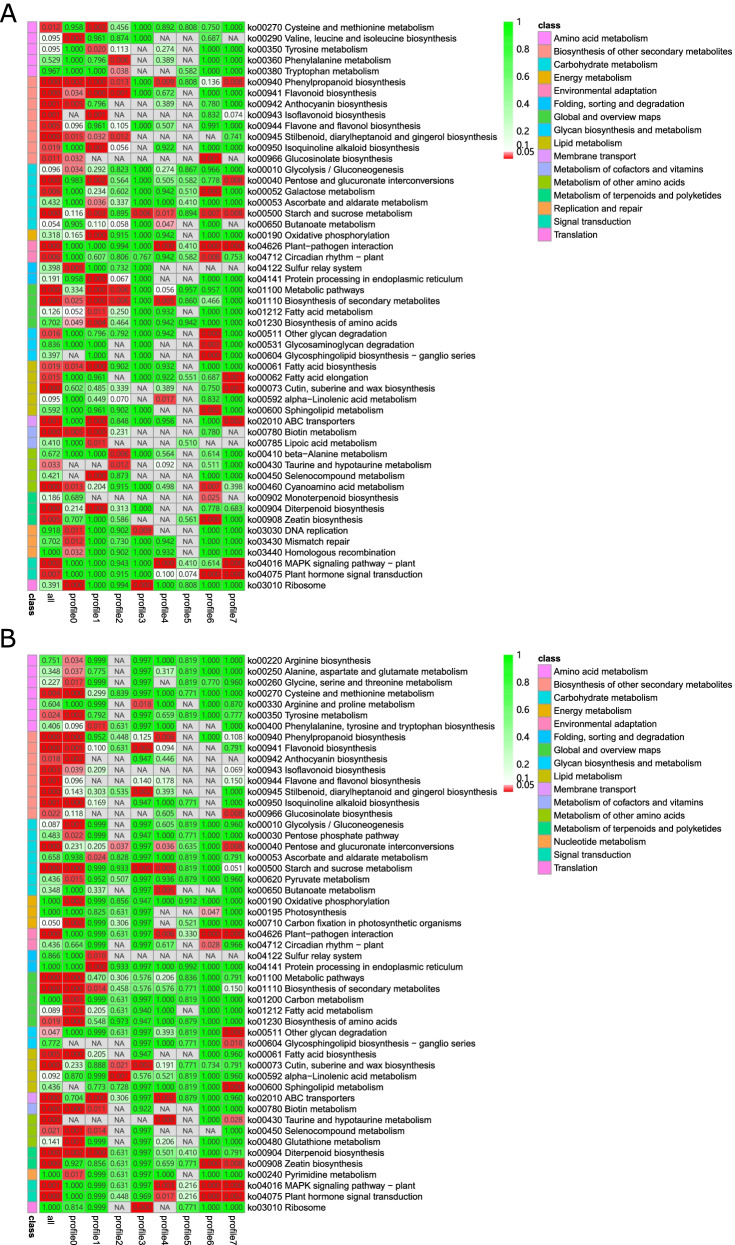
KEGG pathway enrichment analysis for genes in trend profiles of the HTD (**A**) and MTD (**B**) groups. We obtained permission to use the KEGG software from the Kanehisa laboratory. MTD, dodders attached to the stems of non-host plant; HTD, dodders attached to the stems of host plant

To assess gene expression patterns during haustoria development, 12 919 genes in the HTD and 12 900 genes in the MTD were separately clustered into eight profiles, in which profiles 0, 1, and 7 were common among the two types of samples significantly enriched by genes (Fig. S3). The majority of functional annotation items were generated by genes with significant profiles in each type of sample, which was similar to the functional annotation analysis for metabolites (Fig. 7). In all, 13 KEGG pathways were specific for genes in the trend profiles of HTD, including valine, leucine, and isoleucine biosynthesis (ko00290) and phenylalanine metabolism (ko00360). We identified 12 unique KEGG pathways associated with MTD. The ko00220 arginine biosynthesis pathway was a common pathway enriched by genes and metabolites in the MTD group, whereas ko00460 cyanoamino acid metabolism was unique for HTD, identified by integrating transcriptome and metabolome temporal analysis results. Carbon fixation in photosynthetic organisms (ko00710) converts light energy into chemical energy, which was a specific pathway for MTD compared to HTD. The number of genes with different gene expression patterns was significantly higher than that of genes with the same gene expression pattern (*P* = 0.0012, chi-square test). However, few DEGs and no DAMs were found between the HTD and MTD in this pathway.

**Fig. 8:**
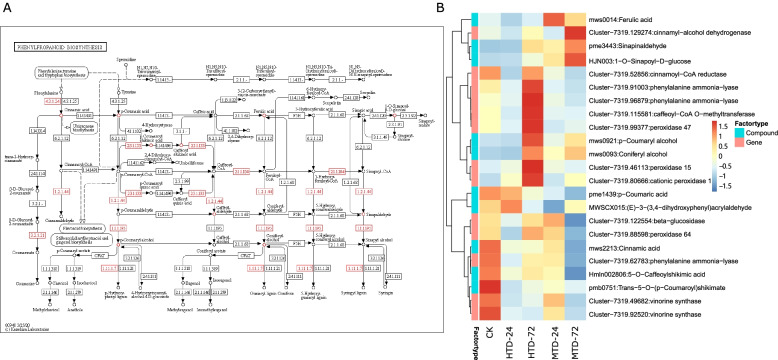
DEGs presented in phenylpropanoid biosynthesis pathway (**A**). Heatmap for DEGs and DAMs in phenylpropanoid biosynthesis pathway (**B**). MTD, dodders attached to the stems of non-host plant; HTD, dodders attached to the stems of host plant; DEGs, differentially expressed genes; DAMS, differentially abundant metabolites

Phenylpropanoids play an important role in plant responses to biotic and abiotic stimuli [51]. They mediate plant resistance to pests. KEGG analysis was used to annotate the up- or downregulated genes and metabolites involved in phenylpropanoid biosynthesis (Fig. 8). The number of genes and metabolites with similar regulation patterns was significantly larger than that of altered genes and metabolites (*P* = 0.0001, chi-square test). Our non-targeted metabolome analysis indicated the highest abundance of p-coumaryl alcohol and coniferyl alcohol in HTD at 72 haa, whereas the accumulation of ferulic acid, sinapaldehyde and 1-O-sinapoyl-D-glucose gradually increased and peaked at 72 haa. Moreover, we observed that the gene expression patterns of DEGs in HTD and MTD were similar to those of the metabolites. The levels of cinnamoyl-CoA reductase, phenylalanine ammonia-lyase, caffeoyl-CoA O-methyltransferase, and peroxidase in HTD were significantly high in the inoculated stems at 24 and 72 ha. The peak expression value of cinnamyl alcohol dehydrogenase appeared in the MTD at 72 haa. These results suggest that DAMs and DEGs present various regulatory trends based on hosts inoculated with *C. japonica*.

## Discussion

### Haustoria of *C. japonica* on host and non-host plants

The haustorium is not only unique to dodders but is also an important organ that enables them to obtain nutrients [50]. The emergence of the haustorium establishes physical and physiological channels between the dodder and its host. The two species are then directly connected at the cellular level, enabling the dodder to obtain the material and energy needed for growth and development through its host plant [22]. Through transcriptome and whole genome sequencing, Wu et al. [54] found that approximately one-third of the highly expressed genes of *C. japonica* are identical to those of autotrophic plant roots, indicating that the evolution of *C. japonica* may be related to changes in the expression of genes involved in autotrophic plant root development [48]. Parasitic plants show strong selectivity for their hosts, which is generally believed to be caused by differences in the host nitrogen content and secondary metabolite synthesis [54]. According to a previous survey on dodder in agricultural and forestry plants in Guangxi, we found that after the stems of *C. japonica* were wrapped around young mango plants, although papillary protrusions (superior haustoria) could be formed at the contact points, endogenous haustoria, where nutrients or water could be transferred, failed to form. In our study, we also observed that the dodder stem became black and eventually died after haustorial connection with non-hosts, in contrast to the green color of dodder stems on binding with host plants, suggesting that nutrition compounds or water could not be transferred from *M. indica* (non-host) to dodder (Fig. 1).

In natural communities, parasitic plants usually parasitize specific plant types [29]. Different parasitic plants choose their hosts in different ways and at different times; however, chemicals play an important role in this process. Some parasitic plants can accurately parasitize host seeds with broken dormancy. This specificity is induced by chemicals released when the host seeds break dormancy and germinate. Other parasitic plants can choose the direction of growth, grow towards the most appropriate host, and finally contact the host to grow haustoria to complete parasitization [44]. Studies have shown that legumes are easily parasitized by parasitic plants because of their high nitrogen content [27]. Kelly [28] found that the number of windings of dodder stems in contact with the host is significantly correlated with the nitrogen content of the host; if the nitrogen content is low, the number of windings is lower, and vice versa. However, plants with low mineral element content or high secondary biomass content are not easily parasitized [29], and the resistance of the host declines with the host plants under stress [18]. In contrast, many studies have shown that most of the induction and recognition of seed germination and haustorium production in obligate parasitic plants occur through allelochemicals that can be used to inhibit adjacent plants during evolution [15]. Secondary metabolites play an important role in the recognition and induction of the three trophic levels of plants, herbivores, and natural enemies. In the present study, metabolomic analysis was performed in the dodders attached to the host plant (HTD) and non-host plant (MTD) to investigate the regulatory mechanism of the interaction between *C. japonica* and host plants. Many metabolites showed different trends and significant differential expression at 24 h and 72 h after parasitizing, revealing that they have different regulatory modes in parasitic hosts (Fig. 2). Thus, the difference in secondary metabolites between hosts is one of the factors underlying host selection in *C. japonica*.

### Fatty acid metabolism in host plants

α-Linolenic acid metabolism and linoleic acid metabolism were significantly enriched pathways with upregulated DAMs in MTD compared with HTD at 24 haa (Fig. 3B), suggesting that fatty acid metabolism may be an underlying mechanism in parasitism. The present study offers new insights into the role of plant lipid pathways in the modulation of plant–pathogen interaction mechanisms, including host resistance. Righetti et al. [45] identified 25 significant metabolites, with glycerophospholipid and linoleic acid metabolism as the main pathways affected by maize-fusarium interactions. Free fatty acid levels increase in response to different stresses and play a crucial role in plant defense [8], and we found that the level of fatty acid metabolism was higher in *C. japonica* in the non-host than in the host at 24 haa (Fig. 3B). Moreover, in contrast to our previous studies, linoleic acid was found to be a marker in the host samples [10, 11]. This suggests that the metabolites produced by the defence system of the non-host mango enter *C. japonica* through material exchange. In contrast, the expression levels of the upstream key gene lipoxygenase (LOX) and downstream key gene acyl-CoA oxidase (ACX) decreased, reducing the consumption of linolenic acid in fatty acid metabolism [13]. *LOX* is a key gene in α-linolenic acid metabolism and regulates the oxidation of α-linolenic acid to form fatty acid hydroperoxide, which is the first step in the conversion of linolenic acid to other substances [31, 55]. ACX is involved in the conversion of alcohols to esters downstream of fatty acid metabolism and is the key rate-limiting enzyme in this pathway [1]. In our study, the expression of LOX was downregulated in the non-host plant compared with that in the host plant (Table S6), which might have promoted the accumulation of α-linolenic acid (Table S2). Similarly, downregulation of ACX expression leads to the accumulation of intermediate products during the metabolic process. Finally, the accumulation of hydroperoxide fatty acids inhibits the catalytic activity of LOX, increasing the content of α-linolenic acid in non-host plants.

### Phenylpropanoid biosynthesis in host plants

Phenylpropanoids play an important role in plant responses to biotic and abiotic stimuli [39]. They mediate plant resistance to pests and pathogens. The phenylpropanoid pathway produces lignin, flavonoids, and other metabolites [56], and the synthesis of lignin-building monolignols is triggered after the cell wall is hit by pathogens [25]. These studies mainly focused on specific gene families related to the phenylpropanoid and monolignol biosynthesis pathways, such as phenylalanine ammonia-lyase (PAL) [21], coniferaldehyde/ferulate 5-hydroxylase (F5H; [30]), cinnamic acid 4-hydroxylase (C4H; [57]), caffeic acid/5-hydroxyferulic acid O-methyltransferase (COMT; [2]), 4-hydroxycinnamoyl-CoA ligase (4CL; [36]), cinnamoyl−CoA reductase (CCR; [16]), cinnamyl alcohol dehydrogenase (CAD; [7]), droxycinnamoyl-CoA:shikimate/quinate hydroxycinnamoyltransferase (HCT; [17]), and caffeoyl-CoA O-methyltransferase (CCoAOMT) [58]. *Cuscuta reflexa* induced tomato cells at the site of infection to secrete soluble phenylpropanol, eliciting a defense program in tomato and showing increased accumulation and activity of peroxidases associated with phenylpropanoids and other components of the cell wall, such as proteins, pectins, or cellulose fibers. The chemical composition of the wound tissue was found to contain substances belonging to the phenylpropanoids, as previously described [37, 38]. In our study, KEGG analysis was used to annotate the up- or downregulated genes and metabolites involved in phenylpropanoid biosynthesis. CCR, PAL, CCoAOMT, and peroxidase levels were significantly higher in the stems at 24 and 72 haa in HTD than in MTD, consistent with previous studies showing that some alterations were positively regulated towards resistance [17]. This suggests a direct link between the induction of parasitism and a surge in the transcription rate of phenylpropanoid pathway genes. Non-host plants might disrupt parasitic processes by inhibiting the phenylpropanoid biosynthesis pathway.

In addition, many prediction methods, including cis-regulatory elements of monolignol biosynthesis pathway genes, have helped reveal the mystery of the transcriptional regulation of phenylpropanoid pathways [33]. Transcriptional regulation of phenylpropanoid biosynthesis has been extensively investigated, and reviews have been published recently [19, 61]. In particular, MYBs are potent transcriptional regulators, and their overexpression or inhibition can lead to dramatic changes in plant phenolic profiles [62]. In tobacco plants, the overexpression of *Pinus taeda* MYB4 resulted in increased expression of C3H, COMT, CCR, CCoAOMT, and CAD; the expression of 4CL and C4H genes was reportedly unaffected, although there was a decline in the expression of PAL genes [43]. In the present study, MYBs were upregulated in host plants at both 24 haa and 72 haa compared with that in non-host plants (Tables S5 and S6), which may be the key reason for the upregulation of pathway genes in phenylpropanoid biosynthesis. The exchange of material between *C. japonica* and its hosts may also be a result of stress responses in host plants.

## Conclusions

This study describes the transcriptomic and metabolomic differences of *C. japonica* when parasitizing host and non-host plants. Complex changes were observed in transcriptome and metabolome regulation suggesting that metabolic pathways, such as α-linolenic acid, linoleic acid metabolism, and phenylpropanoid biosynthesis, might play important roles during haustoria development, and non-host plants might be resistant to dodder through interfering with these metabolic pathways during the formation of haustoria. Related pathway genes such as LOX, ACX, CCR, PAL, and CCoAOMT presented various regulatory trends and might be potential targets for inhibiting haustorium formation. The results provide insights for a better understanding of the metabolic mechanism of dodder–host interactions, and will therefore, contribute to inhibitory activity by identifying and analyzing the structures, functions, and metabolic pathways of differentially expressed metabolites.

## Materials and methods

### Plant material and sampling

All plants were grown in a greenhouse. The plant was identified by Prof. Ma and deposited in the germplasm resources nursery of the Guangxi Academy of Agricultural Sciences (deposition number: tsz-03). Permission to use this line was obtained. All methods were performed in accordance with relevant guidelines and regulations. *Ficus microcarpa* ‘Golden Leaves’ (HTD) and *Mangifera indica* (MTD) plants were cultivated on Jiffy peat pellets under 8 h light and 16 h dark at 22 °C for one month and then individually placed at the bottom of deep pots with a diameter of 16 cm and 14 cm in height. The temperature was maintained at 25 °C (day) and 18 °C (night) under natural light (16 h light, 8 h dark). Dodder seeds were purchased from a traditional Chinese medicinal herb store in Kunming, China. The seeds were dipped in 95% sulfuric acid for 30 min. The treated seeds were then washed 10 times with water and placed on a wet filter paper in the dark at 25 °C, following a previously published method [35]. Four-day-old *Cuscuta japonica* seedlings were attached to the stems of the host (HTD) and non-host (MTD) plants to form growing stocks. For attachment experiments, the plants with similar age, and the stems of host or non-host plants with similar size and close development stage were selected. All stem attachment were carried out at the same time. Normally, the attached position of *C. japonica* would begin to form haustoria and invade the stems of host plant within 24 h post attachment.

To investigate dynamic changes in *C. japonica* during stock growth, proximal stem segments were harvested at 24 h and 72 haa. *C. japonica* usually grows ~ 3 cm per day if it successfully parasite and get nutrients from host plant, and it would grow ~ 1 cm per day without successful parasitism. Therefore, a 2 cm long proximal stem segment of *C. japonica* was cut 1 cm away from the attachment region to host or non-host plants. Three replicates were used for each stage, each of which was pooled from at least five stem segments. We used HTD and MTD strands for the dodder segments captured from the attachment of host-dodders and non-host-dodders, respectively. All specimens were harvested using sterile razor blades, and then frozen in liquid nitrogen and stored at − 80 °C for subsequent metabolomic and transcriptomic analyses.

### Sample preparation and liquid chromatography–mass spectrometry

Fifteen *C. japonica* proximal stem samples including three biological replicates, were used for metabolite preparation. The stem samples were freeze-dried under vacuum and crushed at 30 Hz for 1.5 min using a Scientz-100F lyophilizer (Scientz, China) and an MM 400 grinder (Retsch, Germany) to produce the sample powder. After dehydration and crushing of the samples, approximately 100 mg of each sample powder was weighed and dissolved in 1.2 mL of 70% methanol. The mixtures were vortexed six times, each time for 30 s for 30 min, and stored at 4 °C overnight. The mixtures were then centrifuged at 12 000×*g* for 10 min. We implemented a microporous membrane (pore size:0.02) to filter the supernatant of each sample collected from the extracts in sample bottles for UPLC–MC analysis.

To analyze the extracts, UPLC (SHIMADZU Nexera X2; https://www.shimadzu.com.cn/) and tandem mass spectrometry (MS/MS; Applied Biosystems 4500 QTRAP; http://www.appliedbiosystems.com.cn/) were performed. The liquid phase included an Agilent SB-C18 chromatographic column (2.1 × 100 mm), and the mobile phase, solvent A (0.1% acetic acid in water) and solvent B (0.1% acetic acid in water). The elution gradient program was 0 min with 95% A and 5% B; B linearly increased to 95% in 9 min and maintained at 95% for 1 min, then B dropped 5% from minute 10 to 11.10, and maintained at that ratio from minute 11.1 to 14. An injection volume of 4 μL was loaded onto the column at a flow rate of 0.35 mL/min at 40 °C. The MS parameters were as follows: temperature of the positive electrospray ionization, 550 °C; voltage, 5500 V/− 4500 V; ion source gas I, 50 psi; gas II, 60 psi; curtain gas, 25 psi; collision-activated dissociation, high. Triple quadrupole system (QQQ) scans were acquired using multiple reaction monitoring (MRM) experiments. We further evaluated the declustering potential and collision energy for each MRM ion pair. Finally, the set of MRM transitions was recorded in each period during the elution of the metabolite.

Secondary spectrum information of the metabolites in the *C. japonica* stem samples was qualitatively analyzed by one by one matching with the self-built MetWare database (https://www.metware.cn/). Quantitative analyses of metabolites were performed using MRM. The isotope signal and the repeat signals of K+, Na+, and NH4+ were removed during the quantitative analysis. We computed the mass spectrum peak area integral of all samples and performed an integral correction for each metabolite across all samples. To assess the reliability and reproducibility of the data, we performed quality control by monitoring the changes in the mixtures of the extracted samples.

### Metabolome analysis

Metabolomic analysis was performed using the MetaboAnalystR (1.0.1) package in R [9, 34]. To identify DAMs, we implemented orthogonal partial least squares discriminant analysis (OPLS-DA) using MetaboAnalystR according to the following thresholds: variable importance in projection (VIP) score ≥ 1 and absolute log2 FC ≥ 1. Pathway enrichment analysis of the identified metabolites was performed by mapping them to the KEGG compound database. The significant pathways of DAMs were determined by *P*-values of the hypergeometric test. The PCA was performed using the statistical function prcomp on the R platform.

### RNA extraction and RNA-seq

Total RNA was extracted from *C. japonica* stems from three biological replicates at each stage using the Qiagen RNeasy Plant Kit (Hilden, Germany) according to the manufacturer’s protocol. DNA contamination, quality, concentration, and integrity of the total RNA were confirmed using agarose gel electrophoresis, a NanoPhotometer, qubit 2.0 fluorometer, and Agilent 2100 BioAnalyzer.

The *C. japonica* stem RNA-seq libraries were prepared using the Illumina TruSeq RNA Sample Prep Kit following the manufacturer’s instructions, and the quality of the library was detected using Qubit2.0 and Q-PCR. The cDNA library products that passed quality tests were sequenced on an Illumina HiSeq-2500 platform.

### Transcriptome analysis

To obtain high-quality clean reads, read sets obtained from dodder stems were subjected to adapter removal and quality analysis using CASAVA (1.8.2, Illumina). Read sets with N content exceeding 10% of the number of read bases were considered low-quality sequences and filtered out. The Trinity software package was used for the efficient and robust de novo assembly of clean reads. All read pairs from *C. japonica* retained after the filtering pipeline described above were used for de novo transcriptome assembly using Trinity (2.6.6) with default parameters, performed in a Linux cluster with 48 computing nodes and 1.5 TB memory to construct the unigenes.

The unigenes were functionally annotated and classified using different databases, including nr protein, Swiss-Prot, KEGG, Trembl, Gene Ontology (GO), and Clusters of Orthologous Groups of proteins (COG), using BLAST software. First, we selected the nr, Swiss-Prot, KEGG, and COG databases to confirm sequence directions. Then, the alignment of unigenes and protein databases was implemented using BLASTx. Finally, the protein sequences of unigenes with the highest similarity were retrieved for functional annotation and classification.

Clean reads from *C. japonica* were aligned to assemble the transcripts using Botwie2, and the quantity of gene expression was calculated by RSEM (RNA-seq by Expectation Maximization). The expression value of each unigene was normalized to fragments per kilobase of transcript per million fragments mapped reads (FPKM). To identify differentially expressed genes, we used the DEseq2 package (1.22.2) in R to analyze unstandardized read count data between two samples based on a false discovery rate (FDR) < 0.05, and absolute log2 FC ≥ 1.

### Temporal analysis

Short time-series expression miner (STEM) software can process short time-series data for clustering and statistical biological explanations using exclusive approaches and integrating them with GO and KEGG databases [14]. We used the STEM algorithm with default parameters to analyze trends in the changes in the gene expression profiles of *C. japonica* during the formation of a haustorial connection. The DEGs of *C. japonica* were clustered according to their *P*-values. Clustered profiles with *P* ≤ 0.05 were considered differentially expressed. Genes within the selected clusters were enriched in GO terms and KEGG pathways for functional annotation using the hypergeometric distribution test. Functional items of each selected cluster with Q-values ≤0.05 were retained.

## Supplementary Information

**Additional file 1: Figure S1.** Comparison of metabolite abundance in *Cuscuta japonica* attached to the host and non-host plants at 24 h (A) and 72 h (B). **Figure S2.** Number of differentially clustered metabolites in the HTD (A) and MTD (B) groups. The metabolite abundance trends of the differentially clustered profiles in the HTD (C) and MTD (D) groups are shown. **Figure S3.** Number of differentially clustered genes in the HTD (A) and MTD (B) groups. The gene expression trends of the differentially clustered profiles in the HTD (C) and MTD (D) groups are shown. **Table S1.** Metabolome profiles in *Cuscuta japonica* in the HTD and MTD groups. **Table S2.** Metabolite differences in *Cuscuta japonica* in MTD vs. _HTD at 24 haa. **Table S3.** Metabolite differences of *C. japonica* in MTD vs. _HTD at 72 haa. **Table S4.** Statistic of RNA-seq data. **Table S5.** DEGs of MTD vs HTD at 24 haa. **Table S6.** DEGs of MTD vs HTD at 72 haa.

## Data Availability

The sequencing data generated in the study are deposited to the NCBI SRA database under Bioproject No. PRJNA794948 (revewer’s link: https://dataview.ncbi.nlm.nih.gov/object/PRJNA794948?reviewer=2vmrgeo555smvnimm2pbrhc4fu).

## Abbreviations

ACX: Acyl-CoA oxidase
CAD: Cinnamyl alcohol dehydrogenase
CCoAOMT: Caffeoyl-CoA O-methyltransferase
CCR: Cinnamoyl−CoA reductase
COMT: 5-hydroxyferulic acid O-methyltransferase
DAM: Differentially abundant metabolite
DEG: Differentially expressed gene
haa: Hours after attachment
HTD_24h: Dodder attached to host for 24 h
HTD_72h: Dodder attached to host for 72 h
LOX: Lipoxygenase
MRM: Multiple reaction monitoring
MTD_24h: Dodder attached to non-host for 24 h
MTD_72h: Dodder attached to non-host for 72 h
PAL: Phenylalanine ammonia-lyase
PCA: Principal component analysis
UPLC–MS: Ultraperformance liquid chromatography–mass spectrometry
VIP: Variable importance in projection
