# Supplementary material for: Integrated metabolomic and transcriptomic analyses of the parasitic plant Cuscuta japonica Choisy on host and non-host plants
Source: BMC Plant Biol. 2022 Aug 8;22:393. doi: 10.1186/s12870-022-03773-9 (PMC9358843; doi:10.1186/s12870-022-03773-9)
Supplement: Supplementary file 1 — Additional file 1: Figure S1. Comparison of metabolite abundance in Cuscuta japonica attached to the host and non-host plants at 24 h (A) and 72 h (B). Figure S2. Number of differentially clustered metabolites in the HTD (A) and MTD (B) groups. The metabolite abundance trends of the differentially clustered profiles in the HTD (C) and MTD (D) groups are shown. Figure S3. Number of differentially clustered genes in the HTD (A) and MTD (B) groups. The gene expression trends of the differentially clustered profiles in the HTD (C) and MTD (D) groups are shown. Table S1. Metabolome profiles in Cuscuta japonica in the HTD and MTD groups. Table S2. Metabolite differences in Cuscuta japonica in MTD vs. _HTD at 24 haa. Table S3. Metabolite differences of C. japonica in MTD vs. _HTD at 72 haa. Table S4. Statistic of RNA-seq data. Table S5. DEGs of MTD vs HTD at 24 haa. Table S6. DEGs of MTD vs HTD at 72 haa. [file 12870_2022_3773_MOESM1_ESM.zip › Supplementary Figures.pptx]

## Slide 1
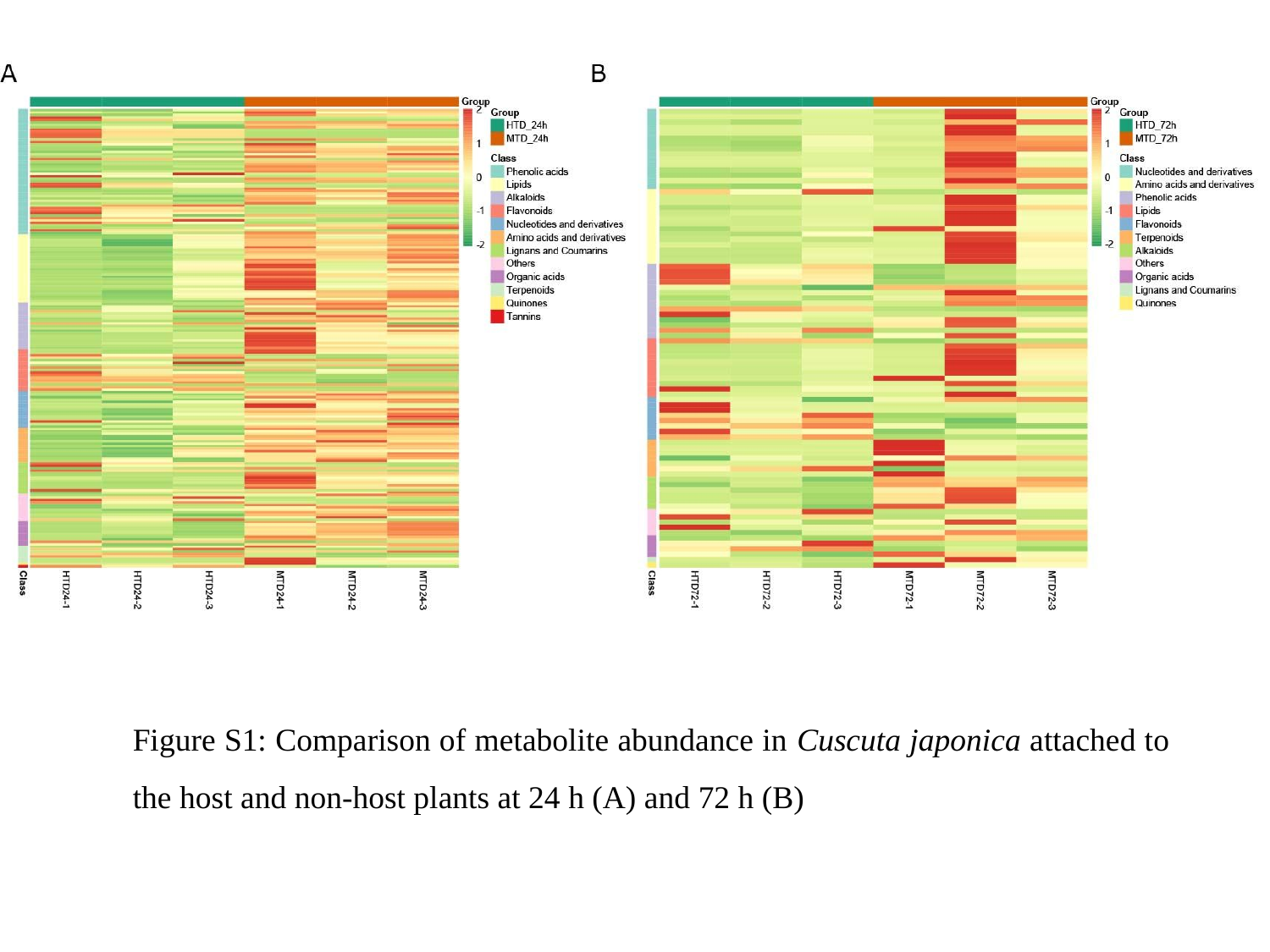

Figure S1: Comparison of metabolite abundance in Cuscuta japonica attached to the host and non-host plants at 24 h (A) and 72 h (B)

## Slide 2
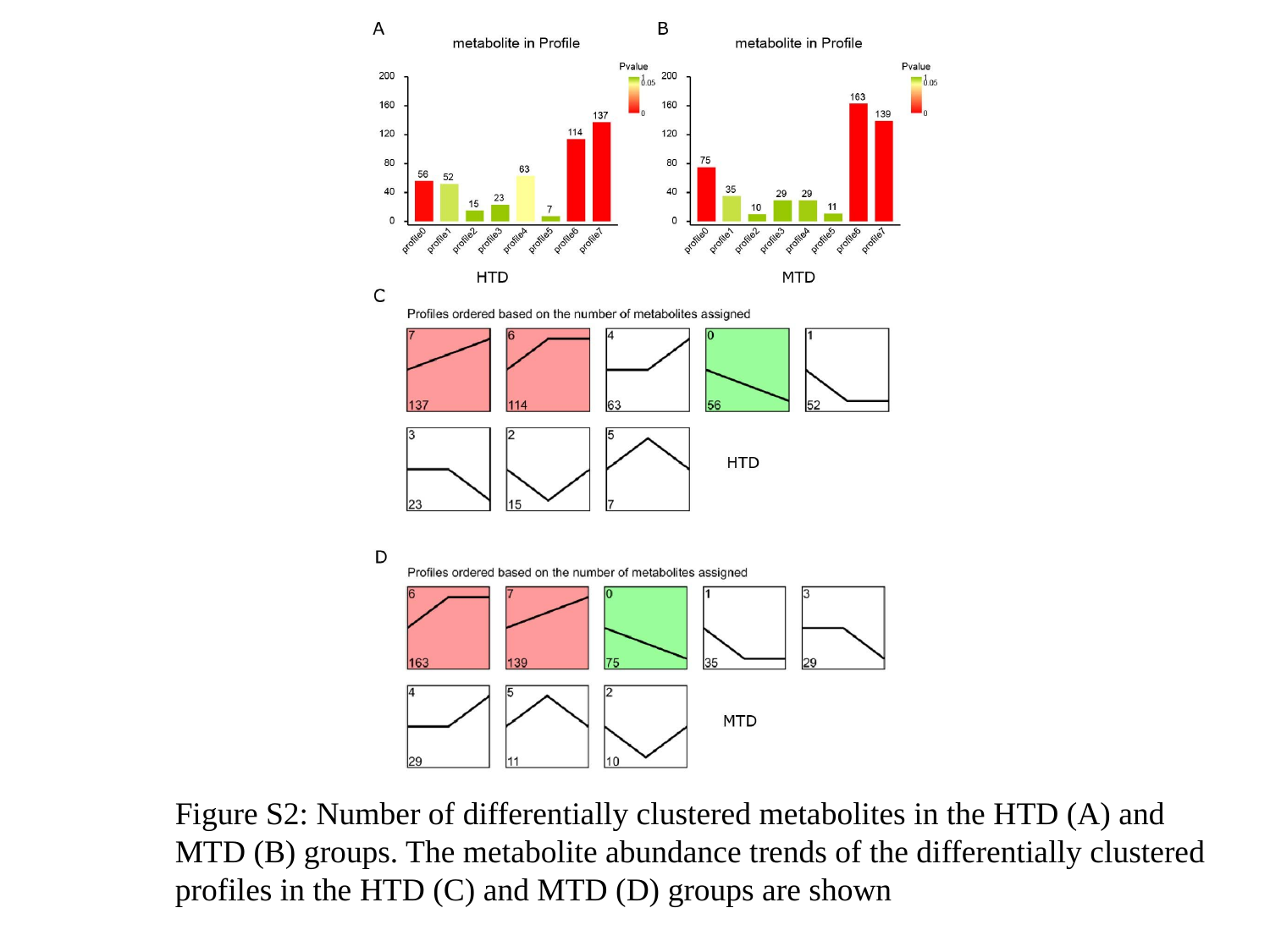

Figure S2: Number of differentially clustered metabolites in the HTD (A) and MTD (B) groups. The metabolite abundance trends of the differentially clustered profiles in the HTD (C) and MTD (D) groups are shown

## Slide 3
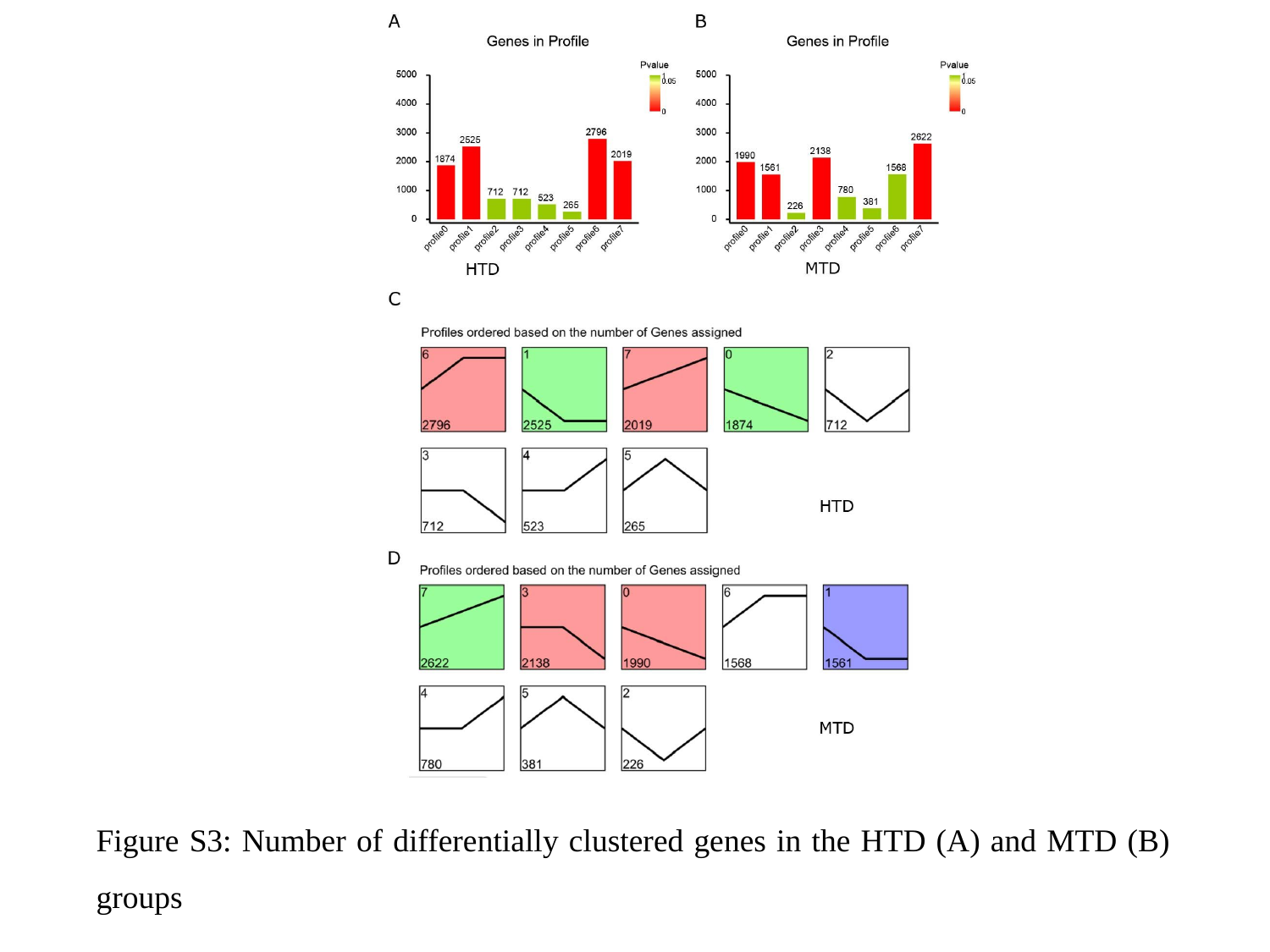

Figure S3: Number of differentially clustered genes in the HTD (A) and MTD (B) groups
